# Fibroblast growth factor 13-mediated regulation of medium spiny neuron excitability and cocaine self-administration

**DOI:** 10.3389/fnins.2023.1294567

**Published:** 2023-11-30

**Authors:** Nolan M. Dvorak, Jessica Di Re, Tileena E. S. Vasquez, Mate Marosi, Poonam Shah, Yorkiris M. Mármol Contreras, Matteo Bernabucci, Aditya K. Singh, Jariatu Stallone, Thomas A. Green, Fernanda Laezza

**Affiliations:** Department of Pharmacology and Toxicology, University of Texas Medical Branch, Galveston, TX, United States

**Keywords:** cocaine use disorder, fibroblast growth factor 13, nucleus accumbens shell, medium spiny neurons, voltage-gated Na^+^ channel

## Abstract

Cocaine use disorder (CUD) is a prevalent neuropsychiatric disorder with few existing treatments. Thus, there is an unmet need for the identification of new pharmacological targets for CUD. Previous studies using environmental enrichment versus isolation paradigms have found that the latter induces increased cocaine self-administration with correlative increases in the excitability of medium spiny neurons (MSN) of the nucleus accumbens shell (NAcSh). Expanding upon these findings, we sought in the present investigation to elucidate molecular determinants of these phenomena. To that end, we first employed a secondary transcriptomic analysis and found that cocaine self-administration differentially regulates mRNA for fibroblast growth factor 13 (FGF13), which codes for a prominent auxiliary protein of the voltage-gated Na^+^ (Na_v_) channel, in the NAcSh of environmentally enriched rats (i.e., resilient behavioral phenotype) compared to environmentally isolated rats (susceptible phenotype). Based upon this finding, we used *in vivo* genetic silencing to study the causal functional and behavioral consequences of knocking down FGF13 in the NAcSh. Functional studies revealed that knockdown of FGF13 in the NAcSh augmented excitability of MSNs by increasing the activity of Na_v_ channels. These electrophysiological changes were concomitant with a decrease in cocaine demand elasticity (i.e., susceptible phenotype). Taken together, these data support FGF13 as being protective against cocaine self-administration, which positions it well as a pharmacological target for CUD.

## 1 Introduction

Cocaine use disorder (CUD), which is characterized by the compulsive use of cocaine despite resultantly deleterious consequences, is a ubiquitous public health problem, with 1.5 million people alone in the United States meeting DSM-5 criteria for the disorder (Schwartz et al., 2022). Compounding the issue of its ubiquity, there are currently no FDA approved treatments for CUD (Schwartz et al., 2022). To address this latter difficulty, the identification of new molecular targets that could become the foundation for medication development is a necessary prerequisite.

Environmental factors are important determinants of CUD. Previous studies have found that environmentally enriched conditions versus isolated conditions alter cocaine use in rats, with rats in the latter condition self-administering increased levels of the stimulant (Green et al., 2010). Correlatively, electrophysiological studies have found that medium spiny neurons (MSN) of the nucleus accumbens shell (NAcSh), which are neurons that provide the sole output of the brain structure (Stanton et al., 2019), display increased firing in slices from rats in environmentally isolated versus enriched conditions (Scala et al., 2018). Given the prominent role of the NAcSh in mesocorticolimbic reward circuity (Stanton et al., 2019), the cellular electrophysiological changes and altered cocaine self-administration observed in isolated versus enriched conditions likely have convergent molecular mechanisms that could provide the basis for new pharmacotherapies for CUD.

To elucidate molecular underpinnings that might converge to produce the electrophysiological and behavioral changes observed in environmentally enriched versus isolated conditions, we performed secondary transcriptomic analyses from a previously published data set (Zhang et al., 2016b). These transcriptomic analyses revealed that cocaine differentially affects expression of growth factor 13 (FGF13) mRNA, which codes for a prominent auxiliary protein of the voltage-gated Na^+^ (Na_v_) channel, in the NAcSh of environmentally enriched rats (i.e., resilient behavioral phenotype) compared to environmentally isolated rats (susceptible phenotype). Subsequently, we used *in vivo* genetic silencing to causally investigate the consequences of knocking down FGF13 in the NAcSh on MSN excitability and cocaine-self administration via acquisition, extinction, progressive ratio, and within-sessions dose response paradigms.

## 2 Materials and methods

### 2.1 Transcriptomics of the nucleus accumbens shell

We manually examined the mRNA expression levels of transcripts from our previous RNA-seq analysis of the NAcSh (Zhang et al., 2016a,b) using previously described bioinformatic analyses (Crofton et al., 2021). IPA analysis was performed as previously described (Zhang et al., 2016a).

### 2.2 Animals

Male Sprague–Dawley rats (Harlan, Houston, TX, United States) were obtained at 21 days (electrophysiology) or 225–250 g (behavior) and maintained in a controlled environment (temperature 22°C; relative humidity, 50%; 12 h light/dark cycle) with free access to food and water. The cocaine self-administration studies were conducted during the light cycle, and animals were fully fed (*ad libitum* feeding schedule) during the tests. All surgical and experimental procedures employed were approved by the University of Texas Medical Branch Institutional Animal Care and Use Committee.

### 2.3 Construction of viral vector knocking down FGF13

The AAV2 vector was constructed to knockdown FGF13 as previously described (Hommel et al., 2003; Benzon et al., 2014). Briefly, a 24 nt target sequence (ACACACTTGCAGGCATTG GATTTC) was chosen from the coding region of the rat FGF13 mRNA sequence. The target sequence was chosen and designed such that the sense and antisense sequences were linked by a miR23 loop. The hairpin was synthesized with *XbaI* and *SapI* restriction endonuclease sites, inserted into an AAV2 plasmid expressing GFP, and verified by sequencing. The promoter for the shRNA was the mouse U6 promoter, and the promoter for GFP was the CMV promoter. To determine the knockdown efficiency of the AAV-shFGF13-GFP vector, stem-cell derived neurons were transfected with either a control hairpin (AAV-shCTRL-GFP; (Zhang et al., 2016b)) or AAV-shFGF13-GFP. Forty-eight hours after transfection, neurons were patched to aspirate the cell contents, which was achieved by applying a small negative pressure after entry into the whole-cell configuration. Glass pipettes for RNA extraction (resistance 6–10 MOhm; 1.5 mm O.D.) were filled with a volume of 0.5 μL of intracellular solution (K-gluconate: 129 mM; KCl: 5 mM; MgATP: 4 mM; Na-GTP: 0.3 mM; Na_2_-phosphocreateine: 10 mM; HEPES: 10 mM; EGTA: 0.2 mM; pH = 7.25; osmolarity = 300 mOsm) and contained a RNase inhibitor (New England Biolabs, Ipswich, MA, United States) at a final concentration of 1 U/μL. After mRNA collection, the glass pipette was placed in a custom-built device (a syringe + PVC flow control + pipette holder) to hold the glass pipette and eject its contents into the 0.2 mL PCR collecting tubes. RNA samples for real time analysis were then quantified using a Nanodrop Spectrophotometer (Thermo Scientific, Waltham, MA, United States) and qualified by analysis on an RNA Nano chip using the Agilent 2100 Bioanalyzer (Santa Clara, CA, United States). The cDNA synthesis was performed with 0.5 μg or 1 μg total RNA in a 20 μL reaction using the reagents in the Taqman Reverse Transcription Reagents Kit (Carlsbad, CA, United States). The reaction was performed as follows: 25°C (10 min), 48°C (30 min), and 95°C (5 min). q-PCR amplifications (performed in duplicate for each sample) were done using 1 μL of cDNA in a total volume of 20 μL using the iTaq Universal SYBR Green Supermix (Bio-Rad, Hercules, CA, United States). The final concentration of the primers was 300 nM. Relative RT-qPCR assays were performed with 18S RNA gene as a normalizer. All PCR assays were run in the ABI Prism 7500 Sequence Detection System and the conditions are as follows: 50°C (2 min), 95°C (10 min), followed by 40 cycles of 95°C (15 s) and 60°C (1 min). The primer sequences used for FGF13 RNA validation were forward 5′-AGG CCG AGG GTG GTA TCT G-3′ and reverse 5′-AGA TCG GGA GAA CTC CGT GAG-3′—which produced a 160 bp amplicon. The knockdown efficiency of AAV-shFGF13-GFP was ~80% (Supplementary Figure S1), similar to the efficiency of our previous constructs (Benzon et al., 2014; Zhang et al., 2016b, 2019; Crofton et al., 2017). The shFGF13 and shCTRL (Zhang et al., 2016b) plasmids were sent to the UNC Vector Core for packaging into the AAV2 capsid. Viral titer was determined using dot blot analysis and ranged from 1×10^10.2^–1×10^12^.

### 2.4 Stereotaxic surgery

To knockdown FGF13, rats were anesthetized with isoflurane (VetEquip, Pleasanton, CA, United States) and injected bilaterally with a control vector (AAV-shCTRL-GFP) or the vector designed to knockdown FGF13 (AAV-shFGF13-GFP) into the NAcSh (1 μL/side over 10 min) using stereotaxic coordinates as previously described (Crofton et al., 2017). For electrophysiology, rats were injected at 21 days old (coordinates: AP = 1.5, *L* = 1.8, *V* = −5.9, 10° lateral angle), and for behavior, rats were injected at 225–250 g (coordinates: AP = 1.6, *L* = 2.2, *V* = −6.7, 10° lateral angle) as previously described (Crofton et al., 2017). Accurate placements were confirmed by extraction of the brain and visualization of GFP using a Dual Fluorescent Protein Flashlight and VG2 barrier filter glasses (Nightsea, Bedford, MA, United States) (Anastasio et al., 2014). For electrophysiological studies, recordings were performed only in GFP expressing cells in the NAcSh by visual identification with a fluorescent microscope. For behavioral studies, placement was checked by dissection after the study, and no animals were excluded due to erroneous vector placement. For cocaine self-administration studies, stereotaxic surgery was concurrently performed with catheter surgery, which was performed as previously described (Crofton et al., 2017, 2021). Catheter patency was maintained by daily flushing with 0.1 mL of heparinized (10 U/mL) saline with ticarcillin (0.067 g/mL).

### 2.5 Whole-cell voltage-clamp recordings

Whole-cell voltage-clamp recordings were performed in MSNs in acute 300 μM coronal brain slice preparations that were prepared as previously described (Tapia et al., 2020). After brain slice preparation, slices were transferred to a recovery chamber containing continuously oxygenated (mixture of 95% O_2_/5% CO_2_) and heated (31°C) artificial cerebrospinal fluid (aCSF) comprised of the following salts: 123.9 mM NaCl; 3.1 mM KCl; 10 mM glucose; 1 mM MgCl_2_; 2 mM CaCl_2_; 24 mM NaHCO_3_; and 1.16 mM NaH_2_PO_4_ (pH = 7.4 and osmolarity = 300–310 mOsm; all salts were purchased from Sigma-Aldrich, St. Louis, MO, United States). After allowing ample recovery, slices were transferred to a recording chamber perfused with aCSF, with the caveat that the aCSF was supplemented with 120 μM CdCl_2_ to block Ca^2+^ currents. For voltage-clamp recordings, borosilicate glass pipettes (Harvard Apparatus, Holliston, MA, United States) with resistance of 1.5–3 MΩ filled with an internal solution comprised of the following salts were used: 100 mM Cs-gluconate (Hello Bio Inc., Princeton, NJ, United States); 10 mM tetraethylammonium chloride; 5 mM 4-aminopyridine; 10 mM EGTA; 1 mM CaCl_2_; 10 mM HEPES; 4 mM Mg-ATP; 0.3 mM Na_3_-GTP; 4 mM Na_2_-phosphocreatine; and 4 mM NaCl (pH = 7.4 and osmolarity = 285 ± 5 mOsm/L; CsOH used to adjust pH and osmolarity; all salts except Cs-gluconate purchased from Sigma-Aldrich). After GΩ seal formation and entry into the whole-cell configuration, a cocktail of synaptic blockers (20 μM bicuculline, 20 μM NBQX, and 100 μM AP5; synaptic blockers purchased from Tocris, Bristol, United Kingdom) was perfused and two voltage-clamp protocols previously described were employed (Dvorak et al., 2021; Marosi et al., 2022). Briefly, to assess the current–voltage relationship of *I*_NaT_ elicited by MSNs and activation properties of *I*_NaT_, MSNs were subjected to voltage commands ranging from −90 mV to +30 mV (Δ = 5 mV) following a 5 ms pre-pulse at −35 mV to mitigate space clamp issues as previously described (Milescu et al., 2010). To assess the inactivation properties of *I*_NaT_ of MSNs, a three-pulse protocol was employed. Following a pre-pulse at −35 mV to mitigate space-clamp issues and returning to the holding potential (−90 mV), cells were stepped to potentials between −100 mV and 0 mV (Δ5 mV) prior to a test potential at −20 mV to assess the voltage-dependence of steady-state inactivation, as previously described (Bosch et al., 2015). The acquired voltage-clamp data was then analyzed as previously described (Dvorak et al., 2021).

### 2.6 Whole-cell current-clamp recordings

Whole-cell current-clamp recordings were performed similar to voltage-clamp experiments, except CdCl_2_ was not added to the superfusing solution and the intracellular solution was comprised of the following salts: 145 mM K-gluconate; 2 mM MgCl_2_; 0.1 mM EGTA; 2.5 mM Na_2_ATP; 0.25 mM Na_2_GTP; 5 mM phosphocreatine; and 10 mM HEPES (pH = 7.2 and osmolarity = 290 mOsm; all salts were purchased from Sigma-Aldrich). For current-clamp recordings, pipette resistance was between 3–5 MΩ. After entry into the whole-cell configuration, the amplifier was switched to *I* = 0 mode for 1–2 min to assess resting membrane potential (RMP), during which time the cocktail of synaptic blockers described above was perfused. After determination of RMP, the amplifier was switched to current-clamp mode, and a previously described protocol designed to assess intrinsic excitability was employed, with collected data being analyzed as previously described (Dvorak et al., 2021).

### 2.7 Immunohistochemistry

Fifteen micrometer slices were prepared from brain tissue previously frozen in liquid nitrogen vapors and then slide mounted and washed with 1× PBS for 5 min, followed by 7 min cold acetone fixation/permeabilization, as has been described previously for imaging AIS proteins (Alshammari et al., 2016; Di Re et al., 2019). Tissue was washed in 1× PBS (3 times, 10 min each), blocked using a 10% NGS solution (Life Technologies, Carlsbad, CA, United States, #50062Z) for 30 min, and stained using the following primary antibodies overnight at 4°C: anti-FGF13 msIgG2b (Invitrogen, Catalog # MA5-27705) and anti-AnkyrinG msIgG2a (Antibodies Incorporated, Davis, CA, United States, 75-146). Both primary antibodies were diluted in a solution of 3% BSA in PBS with 0.1% Tween-20 at 1:500 for FGF13 and 1:300 for AnkyrinG. Following washes with 1× PBS (3 times, 10 min each), isotype specific Alexa Fluor antibodies (anti-msIgG2b 568, Invitrogen A21144; anti-MsIgG2a A21241 647, Invitrogen #A21450) were diluted at 1:250 in a solution of 3% BSA in PBS with 0.1% Tween-20 and applied for 2 h at room temperature followed by 1× PBS washes (3 times, 10 min each), and they were then stained with 4′,6-diamidino-2-phenylindole (DAPI) for 5 min (Invitrogen D1306) and given a final 1× PBS wash (5 min). Tissues were then rinsed with ddH_2_0 and dried for 10–15 min in a 30°C oven before the coverslips were mounted using ProLong Gold (Thermo Fisher #P36930, Waltham, MA, United States).

For vector placement, the following modifications to the above staining procedure were made: Following a wash with 1× PBS for 5 min, 40 μm sections from previously frozen brains were fixed with 4% PFA for 15 min. Following washes, slices were simultaneously blocked and permeabilized using 10% NGS solution +0.5% Triton for 1 h. Slices were stained with anti-GFP (Aves Lab, Davis, CA, United States GFP-1020) at 1:750. An isotype specific Alexa Fluor antibody was used the next day (anti-Chicken IgY 488, Invitrogen A11039).

Confocal images were acquired with a Zeiss LSM-880 confocal microscope. Multi-track acquisition was done with excitation lines at 405 for DAPI, 561 nm for Alexa 568, and 633 nm for Alexa 647. For AIS protein imaging, a 63× oil immersion objective (1.4 NA) was used and *z*-stacks were collected at *z*-steps of 0.43 μm with a frame size of 1,024 × 1,024 and a pixel dwell time of 1 μs. For injection validation, a 10× objective (0.45 NA) was used to capture tile scans (11 × 11 frames) of *z*-stacks which were collected at *z*-steps of 5.51 μm with a frame size of 1,024 × 1,024 and a pixel dwell time of 0.77 μs.

### 2.8 Cocaine self-administration

Two weeks after stereotaxic injection and catheter surgery, rats were placed in operant chambers (Med-Associates, St. Albans, VT, United States) and allowed to self-administer 0.5 mg/kg/infusion cocaine (NIDA drug supply program) on a fixed ratio (FR1) 4 h session for 7 days to achieve stable responding (>15 infusions per session for 2 sessions). A single press of the active lever resulted in illumination of the house lights, located above the levers, and a 0.1 mL intravenous infusion of cocaine delivered for 5.8 s with a 20 s timeout signaled by illumination of both cue lights. Any rats not achieving stable responding were assisted in lever pressing for an additional 3 days until stably responding without assistance. One animal per group was excluded from subsequent study due to failure to achieve stable responding. Twenty-four hours following the acquisition phase, rats underwent within session extinction for 3 sessions. Rats were placed into self-administration chambers and allowed to press for 0.5 mg/kg/infusion cocaine on an FR1 schedule for 1 h. Immediately after, rats received no infusion for 3 h. One rat per group was excluded from day 1 on extinction due to lines unhooking from catheters. Following within session extinction, rats were placed on progressive ratio (PR) schedule for 0.5 mg/kg/infusion cocaine for 3 sessions. For the PR schedule, each successive reinforcement required an increasing number of lever press responses according to the following semi-logarithmic progression: 1, 2, 4, 6, 9, 12, 15, 20, etc., as previously described (Vasquez et al., 2021). After this, rats underwent a within-sessions dose response (WSDR) paradigm for 3 days, in which the dose of cocaine per infusion is halved every 30 min, starting with 0.5 mg/kg and ending with 0.004 mg/kg for a total of 8 doses of cocaine. For the WSDR, the infusion rate was constant at 0.1 mL over 5.8 s. While the infusion rate was kept constant, the infusion duration was varied. Additionally, at low doses, a different syringe containing a lower concentration of cocaine was used. Thus, the chronology of infusion durations was as follows: 11.6 s, 5.8 s, 2.9 s, 1.45 s, 0.725 s, syringe change, 2.9 s, 1.45 s, and 0.725 s.

### 2.9 Statistics

Student’s *t*-tests were used for electrophysiological experiments to determine differences between MSNs expressing AAV-shCTRL-GFP versus AAV-shFGF13-GFP (*p* < 0.05 was considered statistically significant). For voltage-clamp experiments, *n* = 5 cells total per group were recorded from slices from *N* = 2 rats per group (*n* = 2–3 cells/rat). For current-clamp experiments, *n* = 10 cells total per group were recorded from slices from *N* = 3 rats per group (*n* = 3–4 cells/rat). Electrophysiological experiments used a randomized-based design and analysis was not blinded. Normality was assessed, and electrophysiological data sets displayed a normal distribution. No outliers were removed.

Two-way mixed model ANOVA were used to determine differences in acquisition, extinction, and PR responses in animals injected with AAV-shCTRL-GFP versus AAV-shFGF13-GFP. Normality was assessed and log-normalized if data were not normally distributed. A two-way ANOVA was used to assess differences in WSDR data between animals injected with AAV-shCTRL-GFP versus AAV-shFGF13-GFP. In all cases, *p* < 0.05 was considered significant and normality was assessed. Only the acquisition and PR lever press data required log-normalization and all other data sets displayed normal distribution. No outliers were removed.

### 2.10 Cocaine self-administration—behavioral economics data analysis

Behavioral economics analysis focused on the two classical measures derived from the WSDR as a demand curve: *Q*_0_ (demand intensity; intake when price is minimal) and *α* (demand elasticity; rate consumption decreases as price increases). The formula for *Q*_0_ was log *Q* = log(*Q*_0_) + *k* × (e^(−*αQ*^_0_^*C*)^ − 1) where *Q* is consumption, *Q*_0_ is demand intensity, *C* is unit price, and *α* is demand elasticity (slope of function). The constant *k* was set to 3.5. The first dose (0.5 mg/kg/inf) was eliminated before analysis due to the drug loading confound.

## 3 Results

### 3.1 Cocaine differentially regulates FGF13 mRNA in the NAcSh of enriched vs. isolated rats

**Figure 1:**
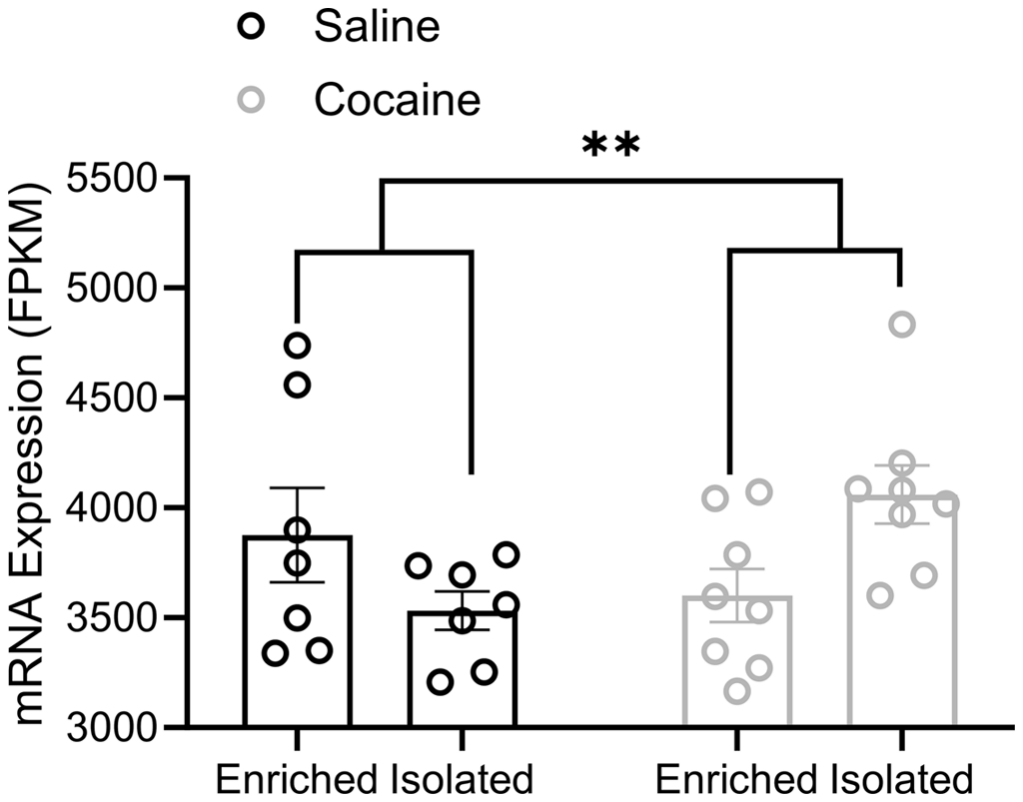
Cocaine differentially regulates FGF13 mRNA levels in environmentally enriched versus environmentally isolated conditions. Bar graph comparing FGF13 mRNA levels in the NAcSh of either environmentally enriched or isolated rats that self-administered saline or cocaine. A significant main effect of cocaine (shown) and an interaction effect (cocaine × housing effect) were found. Data are mean ± SEM (*n* = 7–8 rats/group). Significance was assessed with a likelihood ratio *F*-test (^**^p < 0.01).

To investigate molecular mechanisms that might converge to produce the electrophysiological and behavioral phenotypes observed due to environmental factors and cocaine self-administration, we mined a previous large-scale differential transcriptomic dataset derived from the NAcSh of rats in environmentally enriched or isolated conditions that self-administered either saline or cocaine (Zhang et al., 2016b). An ingenuity pathway analysis found an effect of cocaine self-administration on the FGF Signaling Pathway [−log(*p*-value) = 1.5], as well as an effect of environmental enrichment [−log(*p*-value) = 3.59]. Additionally, cocaine differentially regulated FGF Signaling in enriched vs. isolated rats [interaction −log(*p*-value) = 3.36]. The ingenuity upstream regulator analysis suggests that FGF13 may contribute to cocaine regulation of gene transcription (*p* = 0.02). Analysis of FGF13 mRNA itself showed that it is regulated in the NAcSh by cocaine versus saline self-administration (*p* < 0.01). We observed that there was a trend for environmental enrichment versus isolation in terms of regulating FGF13 mRNA levels in the NAcSh (*p* = 0.09), and that cocaine differentially regulated FGF13 in environmentally enriched versus environmentally isolated rats (*p* < 0.005). Related to the latter, the interaction effect of cocaine and housing condition suggests that cocaine self-administration following environmental enrichment differentially affects the mRNA level of FGF13 compared to their isolated counterparts (Figure 1). On account of this differential regulation of FGF13 mRNA levels in the NAcSh induced by cocaine self-administration in environmentally enriched versus isolated conditions, we elected to further investigate the casual role of FGF13 as it relates to regulating neuronal activity of the NAcSh and cocaine self-administration.

### 3.2 FGF13 in the NAcSh regulates Na_v_ channel function

**Figure 2:**
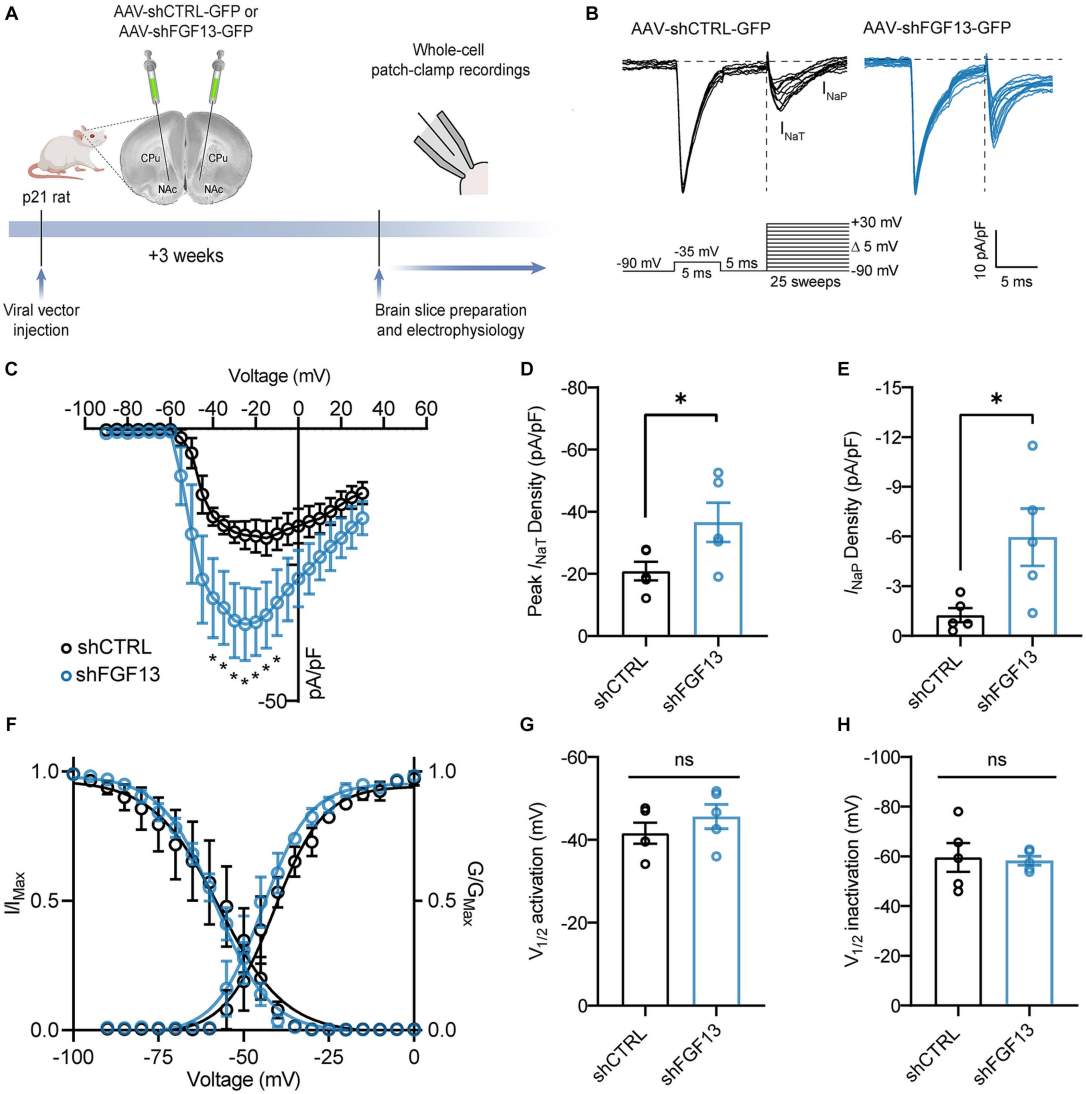
FGF13 regulates the *I*_NaT_ and *I*_NaP_ of MSNs of the NAcSh. **(A)** Schematic of AAV injection site in the NAcSh and experimental timeline. **(B)** Representative traces of sodium current elicited by MSNs expressing AAV-shCTRL-GFP or AAV-shFGF13-GFP in response to the depicted voltage-clamp protocol. **(C)** Current–voltage relationship of the *I*_NaT_ elicited by MSNs belonging to the experimental groups described in **(B)**. **(D)** Comparison of the peak *I*_NaT_ density of MSNs belonging to the indicated experimental group. **(E)** Comparison of the *I*_NaP_ density at the −20 mV voltage command for the indicated experimental groups. **(F)** Comparison of the voltage-dependences of activation and steady-state inactivation of *I*_NaT_ elicited by MSNs of the experimental groups described in **(B)**. **(G,H)** Comparison of V_1/2_ of activation **(G)** and steady-state inactivation **(H)** of *I*_NaT_ of MSNs belonging to the indicated experimental groups. Data are mean ± SEM (*n* = 5 cells/group; slices from *N* = 2 rats/group). Significance was assessed using a student’s *t*-test (ns, not significant; ^*^p < 0.05). For detailed statistical information, refer to Supplementary Table S1.

Given that FGF13 is a prominent Na_v_ channel auxiliary protein (Wittmack et al., 2004; Pablo et al., 2016; Barbosa et al., 2017), we next sought to investigate the role of FGF13 in the NAcSh as it relates to regulating the transient sodium current (*I*_NaT_) and persistent sodium current (*I*_NaP_) of MSNs of the NAcSh, which represent ~95% of the total cell population (Kawaguchi et al., 1995). To do so, rats were stereotaxically injected with either AAV-shCTRL-GFP or AAV-shFGF13-GFP into the NAcSh (Figure 2A). Three weeks after injection, whole-cell voltage-clamp recordings were performed in GFP positive MSNs in slices from the NAcSh. To mitigate space-clamp issues, we adapted a previously described protocol (Milescu et al., 2010) that employs a depolarizing pre-pulse step to inactivate Na_v_ channels distant from the recording electrode, which is followed shortly after by a second step to record Na_v_ channels near to the recording electrode (Alexander et al., 2019) (Figure 2B). Consistent with a previous report showing that knockdown of FGF13 in cultured hippocampal neurons increased the *I*_NaT_ density (Pablo et al., 2016), we found that *in vivo* genetic silencing of FGF13 in the NAcSh augmented the *I*_NaT_ density of MSNs compared to MSNs expressing AAV-shCTRL-GFP (Figures 2C,D). In addition to increasing the peak *I*_NaT_ density, knockdown of FGF13 in the NAcSh increased the *I*_NaT_ density of MSNs at voltages near to the spike threshold (~ −40 mV) compared to MSNs expressing AAV-shCTRL-GFP (Figure 2C).

Given that in a previous report FGF13 was shown to regulate *I*_NaP_ in a heterologous system (Burel et al., 2017), we also investigated if FGF13 regulated the *I*_NaP_ of MSNs of the NAcSh. Consistent with the aforementioned study that found that overexpression of FGF13 in a heterologous system decreased *I*_NaP_ (Burel et al., 2017), we found that *in vivo* genetic silencing of FGF13 increased the *I*_NaP_ density of MSNs compared to MSNs expressing AAV-shCTRL-GFP (Figure 2E). Whereas the *I*_NaT_ and *I*_NaP_ densities were significantly altered due to knockdown of FGF13, the voltage-dependence of activation of *I*_NaT_ (Figures 2F,G) and the voltage-dependence of steady-state inactivation of *I*_NaT_ (Figures 2F,H) were not significantly different between MSNs expressing AAV-shCTRL-GFP versus AAV-shFGF13-GFP.

### 3.3 FGF13 in the NAcSh regulates excitability of MSNs

**Figure 3:**
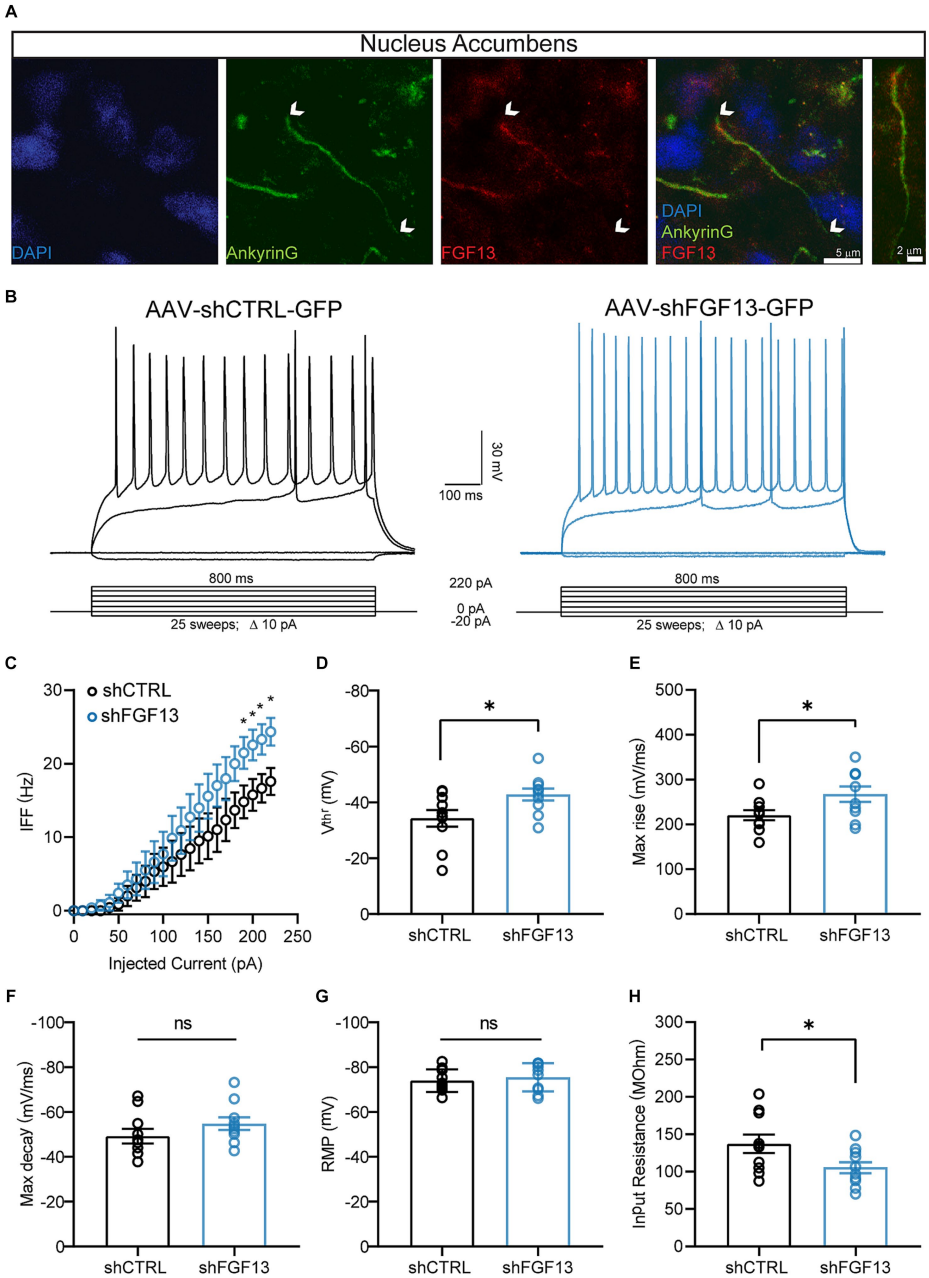
FGF13 regulates the excitability of MSNs of the NAcSh. **(A)** Staining of FGF13 at the AIS. Representative image of DAPI (blue), AnkyrinG (green) and FGF13 (red) at the AIS in the NAcSh with overlay and zoom to AIS. Scale bars indicate 5 μm and 2 μm. **(B)** Representative traces of evoked APs of MSNs expressing AAV-shCTRL-GFP or AAV-shFGF13-GFP in response to the depicted current-clamp protocol. **(C)** Comparison of the IFF of MSNs expressing AAV-shCTRL-GFP (black) or AAV-shFGF13-GFP (blue) over a range of injected current stimuli. **(D–H)** Comparison of the V_thr_ for AP initiation **(D)**, maximum rise of the AP upstroke **(E)**, maximum decay of the AP downstroke **(F)**, RMP **(G)**, and *R*_in_ **(H)** between the indicated experimental groups. Data are mean ± SEM (*n* = 10 cells/group; slices from *N* = 3 rats/group). Statistical significance was assessed using a student’s *t*-test (ns, not significant; ^*^p < 0.05). For detailed statistical information, refer to Supplementary Table S2.

Given that FGF13 displays strong immunoreactivity at the axon initial segment (AIS; Figure 3A), which represents the site for action potential (AP) initiation (Huang and Rasband, 2018), and having shown that knockdown of FGF13 in the NAcSh increases the *I*_NaT_ and *I*_NaP_ densities of MSNs, which are currents that prominently regulate neuronal excitability (Bean, 2007), we hypothesized that *in vivo* genetic silencing of FGF13 in the NAcSh would increase the intrinsic excitability of MSNs. To test this hypothesis, similar to the voltage-clamp experiments described above, rats were stereotaxically injected with either AAV-shCTRL-GFP or AAV-shFGF13-GFP into the NAcSh. Three weeks after injection, whole-cell current-clamp recordings were performed in GFP positive MSNs in slices.

Consistent with the effects of FGF13 knockdown on the *I*_NaT_ and *I*_NaP_ densities of MSNs, *in vivo* genetic silencing of FGF13 resulted in MSNs displaying increased excitability compared to MSNs expressing AAV-shCTRL-GFP (Figure 3B). Quantitatively, knockdown of FGF13 resulted in MSNs displaying increased instantaneous firing frequencies (IFF) over a range of injected current stimuli compared to MSNs expressing AAV-shCTRL-GFP (Figure 3C). Relatedly, consistent with FGF13 knockdown increasing the *I*_NaT_ density at voltages near to the spike threshold (Platkiewicz and Brette, 2010), MSNs expressing AAV-shFGF13-GFP displayed a hyperpolarized voltage threshold (*V*_thr_) for AP initiation compared to MSNs expressing AAV-shCTRL-GFP (Figure 3D). Related to AP kinetics, knockdown of FGF13 increased the maximum rise of the upstroke of the AP compared to MSNs expressing AAV-shCTRL-GFP (Figure 3E), which is a property primarily regulated by the activity of Na_v_ channels (Bean, 2007; Catterall, 2017), without affecting the maximum decay of the downstroke of the AP (Figure 3F). Related to passive electrical properties, knockdown of FGF13 in the NAcSh did not affect the resting membrane potential (RMP) of MSNs (Figure 3G), whereas MSNs expressing AAV-shFGF13-GFP did display a decreased input resistance (*R*_in_) compared to MSNs expressing AAV-shCTRL-GFP (Figure 3H), an electrophysiological property that is primarily regulated by a variety of K^+^ channels (Pablo and Pitt, 2017; Konakov et al., 2022). Collectively, these current-clamp results point toward knockdown of FGF13 augmenting excitability of MSNs primarily through upregulation of Na_v_ channels, with potentially some effects on K^+^ channel conductances as well.

### 3.4 Knockdown of FGF13 in the NAcSh decreases cocaine demand elasticity

Previously, we found that environmental enrichment leads to a protective form of intrinsic plasticity of MSNs in the NAcSh, as evidenced by MSNs in slices from environmentally enriched rats displaying reduced *I*_NaP_ and intrinsic firing compared to MSNs from environmentally isolated rats (Scala et al., 2018). At the behavioral level, these changes in intrinsic plasticity of MSNs have been shown to correlate with changes in cocaine self-administration, as rats in environmentally enriched conditions show decreases in cocaine self-administration during the acquisition, maintenance, and extinction phases compared to environmentally isolated rats (Green et al., 2010). Given the effects of knocking down FGF13 in the NAcSh on the two aforementioned electrophysiological measurements, we hypothesized that, similar to environmentally enriched versus isolated conditions, the electrophysiological changes could correlate with increased cocaine self-administration.

**Figure 4:**
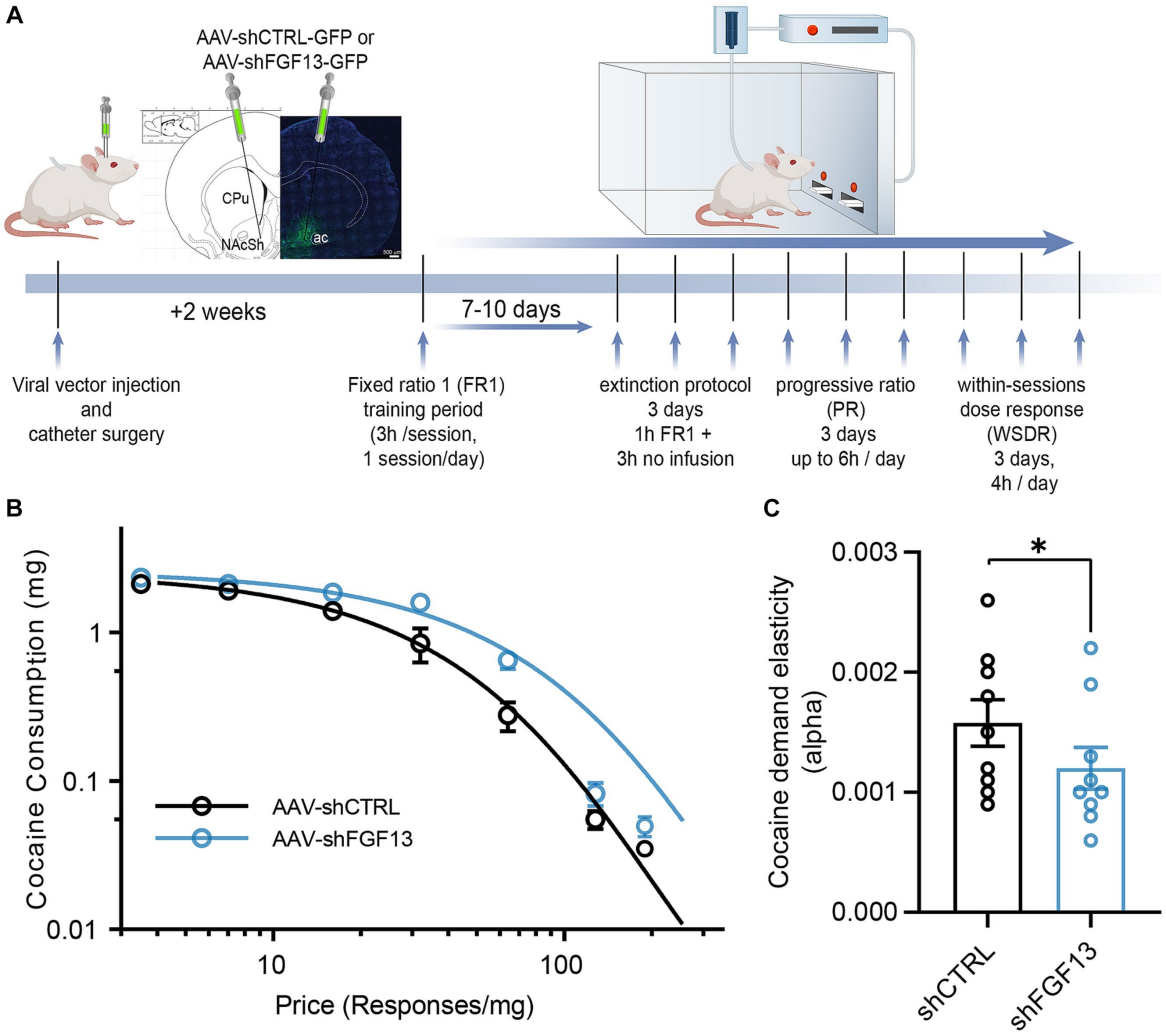
*In vivo* genetic silencing of FGF13 in the NAcSh decreases cocaine demand elasticity. **(A)** Timeline for stereotaxic surgery, catheter implantation, and subsequent cocaine self-administration testing. **(B)** Cocaine demand function across a range of cocaine prices (number of responses for 1 mg/kg of cocaine) for rats belonging to the indicated experimental groups. **(C)** Cocaine demand elasticity (i.e., slope) derived from **(B)** for the indicated experimental groups. Data are mean ± SEM (*n* = 9 rats/group). Statistical significance was assessed using a student’s *t*-test (^*^p < 0.05).

To test this hypothesis, a separate cohort of rats was injected with either AAV-shCTRL-GFP or AAV-shFGF13-GFP into the NAcSh. Two weeks following injection and catheter surgery, rats were placed into operant chambers and self-administered cocaine under a FR1 schedule (Figure 4A). For acquisition of cocaine self-administration (Supplementary Figure S2A), the expected main effect of Session was statistically significant [*F* (6, 98) = 12.15; *p* < 0.01], but no main effect of vector [*F* (1, 18) = 0.5641, *p* = 0.4623] and no interaction [*F* (6, 98) = 0.6702, *p* = 0.6739] were found. Similarly, across three sessions of within-session extinction (Supplementary Figure S2B), the expected main effect of Session was found [*F* (1.634, 24.51) = 44.47, *p* < 0.01], but no effect of vector [*F* (1, 16) = 0.2692, *p* = 0.6110] and no interaction [*F* (2, 30) = 0.05834, *p* = 0.9434] were found. Likewise, for PR reinforcements (Supplementary Figure S2C), there was a similar main effect of session [*F* (1.826, 27.38) = 9.929, *p* < 0.01] but no effect of vector [*F* (1, 16) = 0.09578, *p* = 0.7609] and no interaction [*F* (2, 30) = 0.7143, *p* = 0.4977] were found. PR lever presses (Supplementary Figure S2D) followed the same pattern of a main effect of session [*F* (1.783, 26.74) = 9.725; *p* < 0.01], but no effect of vector [*F* (1, 16) = 0.1485, *p* = 0.7050] or interaction effect [*F* (2, 30) = 0.7022; *p* = 0.5034]. However, for within-session dose response, knockdown of FGF13 in the NAcSh did decrease demand elasticity (*α*) for cocaine without changing consumption at minimal cost (*Q*_0_) (Figures 4B,C), an effect suggesting a susceptible addiction behavioral phenotype.

## 4 Discussion

FGF13 is a member of a group of proteins called intracellular fibroblast growth factors (iFGFs), which comprises FGF11-FGF14 (Zhang et al., 2012). Unlike secreted fibroblast growth factors (FGF1-10 and FGF15-23), which exert diverse effects through extracellular binding to fibroblast growth factor receptors (Zhang et al., 2012), iFGFs are principally known for their direct binding to the intracellular C-terminal domains of Na_v_ channels (Lou et al., 2005; Laezza et al., 2009). Through these direct protein:protein interactions, iFGFs regulate Na_v_ channel kinetics and trafficking to the membrane, which exerts important regulation of neuronal excitability (Pitt and Lee, 2016).

Whereas FGF13 has been extensively studied related to its role in regulating the function of Na_v_ channels in sensory neurons of the peripheral nervous system (Yang et al., 2017; Effraim et al., 2019; Wang et al., 2021), as well as for its role as a microtubule stabilizing protein (Wu et al., 2012), its role in terms of regulating the activity of neurons in the central nervous system (CNS) has received less attention. In one of the few reports to date investigating the role of FGF13-mediated regulation of the activity of CNS neurons, Pablo et al. (2016) found that knockdown of FGF13 in cultured hippocampal neurons increases the *I*_NaT_ density. Consistent with this previous report, we similarly find that knockdown of FGF13 increases the *I*_NaT_ density of MSNs in slices.

While our finding is consistent with this previous report, it is intriguing to note that conditional knockout of FGF13 decreases the *I*_NaT_ density of dorsal root ganglion (DRG) neurons (Yang et al., 2017; Wang et al., 2021). These differential effects of depleting FGF13 in hippocampal and striatal neurons versus DRG neurons likely arise due to differences in Na_v_ channel isoform and iFGF expression in the neuronal populations. For example, Na_v_1.1, Na_v_1.2, and Na_v_1.6 represent the primary Na_v_ channel isoforms expressed in hippocampal and striatal neurons, with Na_v_1.6 being most enriched in MSNs (Scala et al., 2018) and CA1 pyramidal neurons (Royeck et al., 2008), whereas Na_v_1.1 is primarily enriched in interneurons (Ogiwara et al., 2007). Conversely, Na_v_1.7 and Na_v_1.8 represent the predominant Na_v_ channel isoforms expressed in DRG neurons (Hameed, 2019). Furthermore, whereas FGF13 is enriched in MSNs and hippocampal neurons along with FGF14 (Hsu et al., 2016; Ali et al., 2018), expression levels of FGF14 in DRG neurons are quite low (Wang et al., 2021). Based upon these differences, future studies that examine if FGF13’s opposite functional regulation of hippocampal and striatal neurons versus DRG neurons is conferred by differences in Na_v_ channel isoform expression, iFGF interplay, or a combination of both are warranted.

In addition to increasing the *I*_NaT_ density of MSNs, we also found that knockdown of FGF13 increases the *I*_NaP_ density of MSNs, which is consistent with a previous report showing that overexpression of FGF13 in a heterologous system decreases *I*_NaP_ (Burel et al., 2017). As anticipated given these effects of FGF13 knockdown on the *I*_NaT_ and *I*_NaP_ densities of MSNs, we correspondingly found that *in vivo* genetic silencing of FGF13 increased the intrinsic excitability of MSNs. Notably, similar to the voltage-clamp findings, the increase in firing frequency of MSNs caused by *in vivo* genetic silencing is opposite compared to the effect of conditional knockout of FGF13 in DRG neurons (Wang et al., 2021), likely owing to the aforementioned differences in Na_v_ channel isoform and iFGF expression in the neuronal populations. In addition to increasing the IFF across a range of injected current stimuli, knockdown of FGF13 also caused a hyperpolarizing shift in the *V*_thr_ for AP initiation and an increase in the maximum rise of the upstroke of the AP, which represent additional changes in firing properties consistent with modulation of Na_v_ channels (Bean, 2007; Catterall, 2017). Related to passive electrical properties, knockdown of FGF13 also intriguingly decreased the R_in_ of MSNs, which is an electrophysiological property primarily regulated by several K^+^ channels (Pablo and Pitt, 2017; Konakov et al., 2022). Notably, in a previous report Pablo and Pitt (2017) showed that knockdown of FGF14 in cultured hippocampal neurons increased the R_in_, which could indicate that FGF13 and FGF14 confer opposite regulation of K^+^ channel conductances in neurons of the CNS; although, future voltage-clamp studies are warranted to unequivocally support such a hypothesis.

MSNs, which are GABAergic neurons that comprise ~95% of the total cell population of the NAc (Kawaguchi et al., 1995), provide the sole output of the brain structure (Stanton et al., 2019). The remainder of the NAc’s neuronal population is comprised of a variety of GABAergic interneurons, including cholinergic interneurons, parvalbumin interneurons, and somatostatin interneurons (Kawaguchi et al., 1995). MSNs are commonly subdivided as D1 or D2 MSNs based upon predominant expression of the dopamine D1 or D2 receptor (Gong et al., 2003). Related to mesolimbic reward circuitry that is altered in CUD (Nestler and Carlezon, 2006), D1 MSNs predominantly project directly onto the ventral tegmental area (VTA) to alter the structure’s release of dopamine onto the NAcSh, whereas D2 MSNs predominantly project onto GABAergic neurons of the ventral pallidum, which, in turn, synapse onto the VTA and alter the structure’s dopaminergic output (Klawonn and Malenka, 2018). Given that MSNs comprise ~95% of the total cell population of the NAc and provide the sole output of the brain structure, increasing their activity through *in vivo* genetic silencing of FGF13 is expected to cause a significant increase in accumbal release of GABA, which, through altering mesolimbic reward circuitry by modulating the dopaminergic output of the VTA, is expected to contribute to our observed behavioral phenotypes related to cocaine self-administration. Although it is possible that knocking down FGF13 could also affect the activity of different types of NAc interneurons, it is unlikely that such changes would substantially alter the net effect of FGF13 knockdown as it relates to increasing accumbal output of GABA. For example, if *in vivo* genetic silencing of FGF13 also increased the activity of NAc interneurons, which synapse onto NAc MSNs, this could have an inhibitory effect on MSNs due to increasing their GABAergic input. However, this increase in GABAergic input would mostly be overridden by the increase in the intrinsic excitability of MSNs caused by *in vivo* genetic silencing of FGF13. Thus, while effects of *in vivo* genetic silencing of FGF13 on NAc interneurons are possible, effects on interneuron activity related to accumbal output compared to increasing the intrinsic excitability of MSNs are expected to be minimal. Related to non-neuronal cell types, AAV2 has tropism for neurons (Haggerty et al., 2020), making it unlikely that our vector exerts effects on cell types such as glia and astrocytes.

In our previous work, we have shown that environmental enrichment, when compared to environmental isolation, leads to a protective form of intrinsic plasticity, consisting of reduced *I*_NaP_ and intrinsic firing of MSNs of the NAcSh (Scala et al., 2018). In behavioral studies, these electrophysiological changes correlate with a protective behavioral phenotype in relation to cocaine self-administration, as environmentally enriched rats display decreased cocaine self-administration during the acquisition, maintenance, and extinction phases compared to environmentally isolated rats (Green et al., 2010). Given that genetic silencing of FGF13 increased the *I*_NaP_ and excitability of MSNs, we hypothesized that FGF13 in the NAcSh might be protective against cocaine self-administration, and that knockdown of FGF13 would increase cocaine seeking. Consistent with this hypothesis, and in line with our previous studies of environmental enrichment (Green et al., 2010; Scala et al., 2018), GSK3β (Crofton et al., 2017; Scala et al., 2018), CREB (Dong et al., 2006; Larson et al., 2011), and FABP5 (Crofton et al., 2021) linking decreased MSN excitability to a protective behavioral phenotype related to cocaine self-administration, examination of our WSDR data showed that knockdown of FGF13 in the NAcSh decreased cocaine demand elasticity in rats. Although the specific circuital mechanism linking our observed cellular phenotypes and effects on cocaine self-administration is not herein elucidated, a plausible mechanism involves the increase in MSN excitability induced by genetic silencing of FGF13 increasing accumbal GABA release, and, thereby, altering the VTA’s dopamine release onto the NAc via modulatory effects on mesolimbic reward circuitry. Consistent with such a hypothesis, Aberman and Salamone (1999) previously reported that depletion of dopamine in the NAc increased demand elasticity for food reinforces. Thus, future studies that investigate if FGF13 in the NAcSh regulates the dopaminergic output of VTA through modulation of mesolimbic circuitry to control cocaine self-administration are warranted. In addition to FGF13, other transcripts in the FGF signaling pathway that were differentially regulated by cocaine in environmentally enriched conditions versus environmentally isolated conditions included FGFR3, PIK3R3, FGF1, CRKL, PIK3R2, MAP3KI, MAP2K3, CRK, HRAS, PIK3CB, and SOS1. Although not pursued in the present investigation, these as well could confer regulatory effects on neuronal excitability in the NAcSh and influence cocaine self-administration, and, thus, could be the subject of future investigations. One limitation of our study is that electrophysiological experiments were performed in slices from rats in late adolescence, whereas behavioral studies were performed in adult rats. Thus, future studies are warranted to confirm that the electrophysiological changes observed in late adolescence are maintained in adulthood. A second limitation is that for electrophysiological experiments, recordings were performed in MSNs in slices from 2–3 rats per group. Thus, future studies aimed toward reproducing and confirming our electrophysiological findings are warranted.

## 5 Conclusion

Overall, using a combination of transcriptomic analyses, *in vivo* genetic silencing, patch-clamp electrophysiology, and a cocaine self-administration paradigm, we collectively showed that FGF13 in the NAcSh plays a protective role against cocaine self-administration and that these behavioral phenotypes correlate with electrophysiological changes in the Na_v_ channel activity and excitability of MSNs. Based upon these findings, pharmacological approaches that seek to increase the complex assembly of FGF13 and Na_v_ channels to decrease the *I*_Na_ and excitability of MSNs in the NAc could represent a promising strategy for the development of novel therapeutics for CUD.

## Data availability statement

The raw data supporting the conclusions of this article will be made available by the authors, without undue reservation.

## Ethics statement

The animal study was approved by the University of Texas Medical Branch Institutional Animal Care and Use Committee. The study was conducted in accordance with the local legislation and institutional requirements.

## Author contributions

ND: Conceptualization, Data curation, Formal analysis, Investigation, Methodology, Visualization, Writing – original draft, Writing – review & editing. JD: Conceptualization, Data curation, Formal analysis, Investigation, Methodology, Visualization, Writing – review & editing. TV: Conceptualization, Investigation, Methodology, Writing – review & editing. MM: Data curation, Investigation, Methodology, Visualization, Writing – review & editing. PS: Conceptualization, Data curation, Investigation, Methodology, Writing – review & editing. YC: Formal analysis, Methodology, Visualization, Writing – review & editing. JS: Data curation, Investigation, Methodology, Writing – review & editing. TG: Conceptualization, Funding acquisition, Investigation, Methodology, Project administration, Resources, Supervision, Writing – original draft, Writing – review & editing. FL: Conceptualization, Funding acquisition, Project administration, Resources, Supervision, Visualization, Writing – original draft, Writing – review & editing. MB: Data curation, Formal analysis, Methodology, Writing – review & editing. AS: Data curation, Formal analysis, Methodology, Validation, Writing – review & editing.
