# Supplementary material for: Fibroblast growth factor 13-mediated regulation of medium spiny neuron excitability and cocaine self-administration
Source: Front Neurosci. 2023 Nov 30;17:1294567. doi: 10.3389/fnins.2023.1294567 (PMC10720079; doi:10.3389/fnins.2023.1294567)
Supplement: Supplementary file 1 [file Presentation_1.pdf]

Supplementary Material

1 Supplementary Figures and Tables

1.1 Supplementary Tables

Supplementary Table 1

Condition	Peak I_{NaT} Density (pA/pF)	Peak I_{NaP} Density (pA/pF)	$V_{1/2}$ of Activation (mV)	$V_{1/2}$ of Inactivation (mV)
AAV-shCTRL	-20.9 ± 3.0 (5)	-1.25 ± 0.43 (5)	-41.6 ± 2.5 (5)	-59.6 ± 5.8 (5)
AAV-shFGF13	-36.6 ± 6.3[a] (5)	-5.95 ± 1.72[b] (5)	-45.6 ± 2.9 (5)	-58.3 ± 1.8 (5)

Supplementary Table 1: *In vivo* genetic silencing of FGF13 in the NAcSh increases Na_v channel activity of MSNs. Table summary of voltage-clamp data shown in Figure 2. Data are mean ± SEM (*n*).

[a] $p = 0.0273$; AAV-shCTRL vs AAV-shFGF13; Student's t-test.

[b] $p = 0.0293$; AAV-shCTRL + AAV-shFGF13; Student's t-test.

Supplementary Table 2

Condition	IFF at 220 pA (Hz)	V_{thr} (mV)	Max Rise (mV/ms)	Max Decay (mV/ms)	RMP (mV)	R_{in} (MΩ)
AAV-shCTRL	17.6 ± 1.8 (10)	-34.3 ± 3.0 (10)	220.7 ± 11.3 (10)	-49.2 ± 3.2 (10)	-74.0 ± 1.6 (10)	137.3 ± 12.4 (10)
AAV-shFGF13	24.3 ± 1.9 (10)[a]	-42.8 ± 2.2 (10)[b]	267.6 ± 17.2 (10)[c]	-54.8 ± 2.8 (10)	-75.5 ± 2.0 (10)	105.8 ± 8.0[d]

Supplementary Table 2: *In vivo* genetic silencing of FGF13 in the NAcSh increases the intrinsic excitability of MSNs. Table summary of current-clamp data shown in Figure 3. Data are mean ± SEM (*n*).

[a] $p = 0.0194$; AAV-shCTRL versus AAV-shFGF13; Student's t-test.

[b] $p = 0.0333$; AAV-shCTRL versus AAV-shFGF13; Student's t-test.

[c] $p = 0.0350$; AAV-shCTRL versus AAV-shFGF13; Student's t-test.

[d] $p = 0.0472$; AAV-shCTRL versus AAV-shFGF13; Student's t-test.

1.2 Supplementary Figures

Supplemental Figure 1: Validation of AAV-shFGF13-GFP.

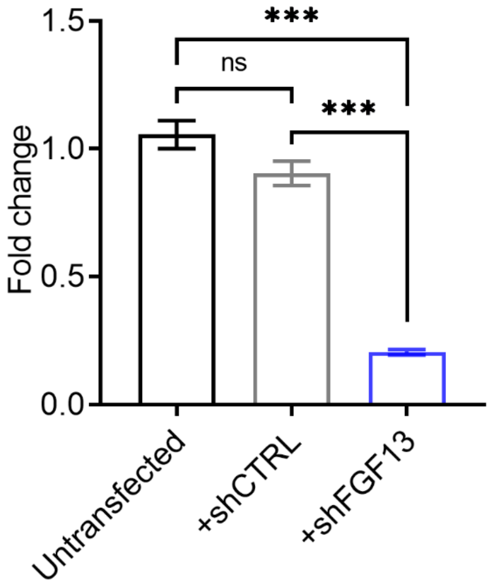

Quantification of FGF13 knockdown efficiency in stem-cell derived neurons that were untransfected, transfected with AAV-shCTRL-GFP, or transfected with AAV-shFGF13-GFP. Data are mean ± SEM (n = 3 biological replicates/group). Statistical significance was assessed using a one-way ANOVA with post hoc Tukey's multiple comparisons test: ns, not significant; ***, $p < 0.001$.

Supplemental Figure 2: *In vivo* genetic silencing of FGF13 in the NAcSh did not alter cocaine acquisition, extinction, or PR.

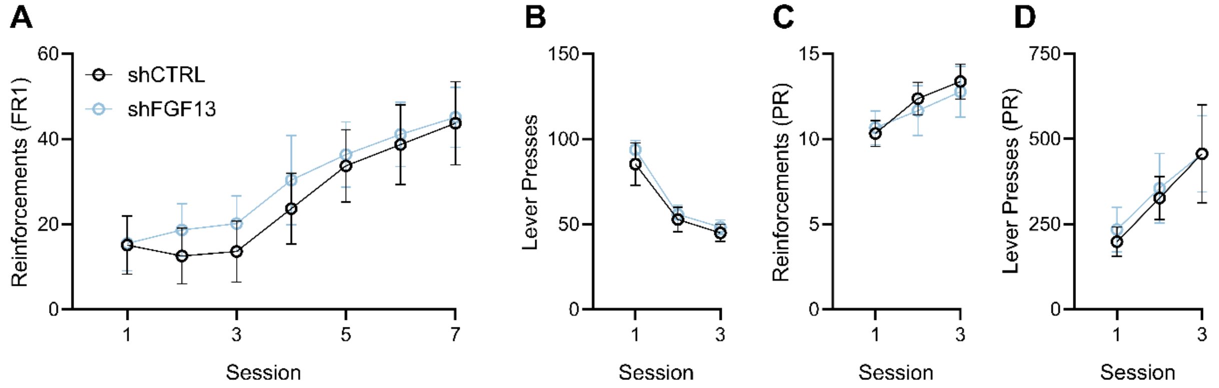

(**A**) Knockdown of FGF13 in the NAcSh did not alter the number of reinforcements of 0.5 mg/kg/infusion of cocaine in a 4 hour FR1 schedule, (**B**) number of lever presses during a 3 hour within session extinction protocol, (**C**) number of reinforcements during a PR schedule, or (**D**) number of lever presses during a PR schedule. Data are mean ± SEM (n = 8-10 rats/group). All data were analyzed via two-way or mixed model ANOVA.
